# Unrest in Infirmary Wards

**Published:** 1920-07-17

**Authors:** 


### Unrest in Infirmary Wards.

On the subject of authorised visitors the medical superintendent has reported to the Lewisham Board of Guardians that every ward contains patients on the danger-list and that rest and quiet are essential to successful treatment. During the morning all the wards are busy with continual traffic of patients coming back from operation, medical officers doing their rounds and dressings, so the patient is continually disturbed. Two afternoons a week (Thursdays and Sundays) are general visiting hours, when the wards are crowded. On other afternoons there are emergency cases and operations, patients to be moved for *x*-ray examination, etc., so that during the ordinary working hours of the day the amount of complete rest that can be given to patients generally is strictly limited. Hence he suggests that authorised visitors be appointed for twelve months, expiring on May 1 in each year; that they shall enter their names in a special Register at the Porter's Lodge; that the visits, precluding religious services, shall be between 2 and 4 p.m. on two days a week. He also urges that no visitors be allowed in the mental wards without the permission of the relatives and of the Medical Superintendent; no visitors in the maternity ward within one week on confinement; and that the number of authorised visitors be limited to four.

